# The Future of Clustered Regularly Interspaced Short Palindromic Repeats (CRISPR)-Cas9 Gene Therapy in Cardiomyopathies: A Review of Its Therapeutic Potential and Emerging Applications

**DOI:** 10.7759/cureus.79372

**Published:** 2025-02-20

**Authors:** Ali Moradi, Sina Khoshniyat, Tochukwu Nzeako, Mohammad Amin Khazeei Tabari, Olawale O Olanisa, Kutiba Tabbaa, Hamza Alkowati, Melika Askarianfard, Daoud Daoud, Olu Oyesanmi, Angelina Rodriguez, Yizhi Lin

**Affiliations:** 1 Internal Medicine, HCA Florida Blake Hospital, University of South Florida (USF) Morsani College of Medicine, Bradenton, USA; 2 Biomedicine, School of Sciences, La Trobe University, Melbourne, AUS; 3 Internal Medicine, Christiana Hospital, Newark, USA; 4 Medicine, Mazandaran University of Medical Sciences, Sari, IRN; 5 Internal Medicine, Trinity Health Grand Rapids, Grand Rapids, USA; 6 Cardiology, HCA Florida Blake Hospital, University of South Florida (USF) Morsani College of Medicine, Bradenton, USA; 7 Medicine, Marmara University, Istanbul, TUR; 8 Family Medicine, HCA Florida Blake Hospital, University of South Florida (USF) Morsani College of Medicine, Bradenton, USA

**Keywords:** arrhythmogenic right ventricular cardiomyopathy (arvc/d), cardiomyopathy, crispr, crispr cas, crispr/cas9 gene editing, crispr-cas9-mediated gene editing, dilated cardiomyopthy, hcm, hypertrophy cardiomyopathy

## Abstract

Cardiomyopathies, among the leading causes of heart failure and sudden cardiac death, are often driven by genetic mutations affecting the heart's structural proteins. Despite significant advancements in understanding the genetic basis of hypertrophic cardiomyopathy (HCM), dilated cardiomyopathy (DCM), and arrhythmogenic right ventricular cardiomyopathy (ARVC), effective long-term therapies remain limited. The advent of clustered regularly interspaced short palindromic repeats (CRISPR) and CRISPR-associated protein 9 (Cas9) gene editing offers a promising therapeutic strategy to address these genetic disorders at their root. CRISPR-Cas9 enables precise modification of pathogenic variants (PVs) in genes encoding sarcomeric and desmosomal proteins, which are frequently implicated in cardiomyopathies. By inducing site-specific double-stranded breaks in DNA, followed by repair through nonhomologous end joining (NHEJ) or homology-directed repair (HDR), this system allows for targeted correction of mutations. In preclinical models, CRISPR-Cas9 has shown promise in correcting HCM-associated mutations in β-myosin heavy chain 7 (MYH7), preventing disease phenotypes such as ventricular hypertrophy and myocardial fibrosis. Similarly, gene editing has successfully rectified DCM-linked mutations in Titin (TTN) and LMNA, resulting in improved heart function and reduced pathological remodeling. For ARVC, CRISPR-Cas9 has demonstrated the ability to repair mutations in desmosomal genes such as plakophilin 2 (PKP2), thereby restoring normal cardiac function and cellular adhesion. Despite these successes, challenges remain, including mosaicism, delivery efficiency, and off-target effects. Nevertheless, CRISPR-Cas9 represents a transformative approach to treating genetic cardiomyopathies, potentially offering long-lasting cures by directly addressing their underlying genetic causes.

## Introduction and background

Cardiomyopathies of genetic origin are among the leading causes of heart failure and sudden death [1]. A large number of hypertrophic cardiomyopathies (HCMs) and at least 30% of dilated cardiomyopathies (DCMs) manifest as familial forms, with the predominant mode of inheritance being autosomal dominant [2]. Cardiomyopathies are genetically heterogeneous, with changes happening at both allelic and nonallelic levels. Familial DCM has been linked to gene mutations encoding sarcomeric proteins and other proteins within the myocyte, including those related to cytoskeletal or nuclear membrane proteins [2]. HCM is a primary cardiac condition defined by left ventricular hypertrophy (LVH) without a clear underlying etiology, with asymmetrical thickening, including the interventricular septum [3]. The genetic background of HCM was originally discussed in the 1990s, with the discovery of pathogenic variants (PVs) in a gene sequence responsible for encoding a sarcomeric protein in a large family with a history of HCM, sudden death, and heart failure [4]. Despite the current understanding of their genetic foundation, there is a huge gap in addressing the medical demand for effective long-term therapy [5]. In recent years, there have been remarkable breakthroughs in cardiac care, mainly due to advancements in genetic therapy. Notably, the development of in vivo clustered regularly interspaced short palindromic repeats (CRISPR) gene editing offers a precise treatment route to permanently repair the mutated genes. The CRISPR-Cas system was initially identified as an adaptive immune system in bacteria and archaeons. Still, it has since been developed as RNA-guided endonucleases (RGENs) for genome editing purposes [6,7]. CRISPR is built upon the foundations laid by earlier protein-guided DNA editing technologies, such as zinc-finger nucleases (ZFNs) and transcription activator-like effector nucleases (TALENs) [8,9]. This method can precisely modify the single-guide RNA (sgRNA) structure without the requirement to create a specific target protein. The CRISPR method consists of two essential components: sgRNA, which is complementary to the desired sequence of DNA, and a CRISPR-associated protein 9 (Cas9) [10]. When target DNA sequences match with the sgRNA in the proximity of a protospacer-adjacent motif (PAM), the Cas9-sgRNA ribonucleoprotein complex commences DNA cleavage. The double-stranded DNA break (DSB) repair process is done using two mechanisms, including nonhomologous end joining (NHEJ), which results in the insertion or deletion (INDELs) of DNA segments, and homology-directed repair (HDR), which properly restores DSBs by incorporating a specific DNA sequence [11]. It is important to note that as postmitotic cells lack HDR machinery, treating genetic cardiomyopathies by gene editing would most likely need NHEJ [10]. CRISPR therapy represents a novel approach to addressing diseases with a genetic background. This study explores the potential of these innovative therapies in treating various types of cardiomyopathies. However, as these approaches are still in their early stages, further research is essential to fully understand their potential applications for patients with these cardiac conditions.

## Review

Genetic basis of cardiomyopathy

Hypertrophic Cardiomyopathy (HCM)

PV in genes producing sarcomeric proteins has been associated with most HCM patients. The most prevalent genes responsible for the disorder are myosin binding protein C (MYBPC3) and β-myosin heavy chain 7 (MYH7), followed by pathogenic mutations in genes producing cardiac troponin T (TNNT2), α-tropomyosin (TPM1), and cardiac troponin I (TNNI3) [12-16]. Less prevalent factors that contribute to HCM include PVs identified in the genes producing cardiac α-actin 1 (ACTC1), myosin light chain 2 (MYL2), myosin light chain 3 (MYL3), and cysteine and glycine-rich protein 3 (CSRP3) [16,17]. MYH7 and MYBPC3 collectively contribute to approximately 50% of all clinically diagnosed cases of HCM and represent at least 75% of affected individuals when PV is detected. In contrast, other genes associated with HCM collectively account for less than 10% of cases [18-20]. Typically, these PVs lead to single-residue substitutions in protein structures that integrate into the sarcomere. However, it's worth noting that nearly half of the documented PVs in MYBPC3 are truncations, and certain MYBPC3 missense variants may lead to haploinsufficiency. Haploinsufficiency occurs when the gene product generated by the wild-type allele is inadequate to compensate for the reduced or absent protein from the variant allele [21,22]. Furthermore, the set of genes related to HCM has broadened to comprise non-sarcomeric genes, including those encoding proteins located in the Z-disk, sarcoplasmic reticulum, and plasma membrane. However, these variations are uncommon, and their function in disease pathogenesis has yet to be studied. Many of these non-sarcomeric gene sequences show substantial genetic correlations, such as disease segregation or in vivo functional data (e.g., CSRP3) [23]. Nonetheless, the role of several of these genes in HCM, such as MYH6, MYLK2, and TCAP, is supported simply by the discovery of variants in diagnosed patients, their absence in controls, and computational analyses [24].

Dilated Cardiomyopathy (DCM)

Despite the well-established genetic foundation for familial DCM, the role of genetics in non-familial DCM remains largely undefined [25,26]. Non-familial, or "idiopathic," DCM is most likely due to either de novo mutations in existing DCM genes or a polygenic contribution from many variants [26-28]. DCM has remarkable genetic variability, involving several cellular proteins and leading to various pathogenic pathways that result in the DCM phenotype. These alterations can impact the structure or function of the sarcomere, nuclear envelope, cytoskeleton, or sarcoplasmic reticulum [27-29]. Herman et al.'s seminal article in 2012 marked a big step forward in understanding the possible significance of genetic processes in DCM. Their findings revealed that uncommon truncating mutations in titin, the biggest protein produced in the heart, account for 15%-25% of DCM cases [30]. TTN spans the sarcomere and is a molecular spring necessary for proper contraction. Different splicing produces different TTN isoforms, with the N2BA, N2A, and N2B isoforms affecting ventricular compliance [31]. Notably, greater expression of the more compliant variants (e.g., N2BA) has been observed in heart failure situations, resulting in left ventricular dilatation [32-34]. Additional genetic contributors of DCM include nonsynonymous uncommon mutations in Lamin (LMNA), which express lamin A and C and constitute around 6% of cases [28]. Loss of LMNA function disturbs cellular signaling, leading to distinct DCM presentations defined by conduction abnormalities and arrhythmias [35]. Notably, frameshift mutations in LMNA confer the greatest risk of sudden cardiac death (SCD) [36]. SCN5A mutations, associated with long QT syndrome and Brugada syndrome, may result in DCM and increase the risk of severe arrhythmias [37]. Another newly found DCM gene is FLNC, expressing filamin C, which, when disrupted, may lead to DCM development [38].

Arrhythmogenic Right Ventricular Cardiomyopathy (AVRC)

ARVC is primarily characterized by the loss of the right ventricular myocardium, which is replaced by fibrous and fatty tissue, potentially leading to arrhythmias by scar-related reentry [39,40]. Biventricular involvement is commonly observed. The underlying process involves mutations in genes producing desmosomal proteins that are involved in cell-to-cell adhesion. This results in myocyte detachment and disrupted intracellular signal transmission [41]. Five key desmosomal genes have been pinpointed as causal factors in ARVC. These include desmoplakin (DSP), desmoglein-2 (DSG2), desmoscollin-2 (DSC2), junctional-plakophilin (JUP), and plakophilin2 (PKP2), the most common among them [42-46]. ARVC, on the other hand, has more genetic complexity than simple monogenic inheritance. Some individuals may have multiple mutations in the same gene (compound heterozygosity) or in separate modifier genes (digenic heterozygosity) [47] (Table 1).

**Table 1:** Genetic basis of cardiomyopathies PVs: pathogenic variants Summarized data of key genes, prevalence, and notes on the genetic causes of hypertrophic cardiomyopathy (HCM), dilated cardiomyopathy (DCM), and arrhythmogenic right ventricular cardiomyopathy (ARVC)

Category	Genetic basis	Key genes	Prevalence	Notes	References
Hypertrophic cardiomyopathy	PVs in sarcomeric protein genes are common	MYBPC3, MYH7	MYBPC3, MYH7: ~50% of cases	MYBPC3 often has truncating variants	[12-14,18-20]
Hypertrophic cardiomyopathy	Less common genes also contribute to HCM	TNNT2, TPM1, TNNI3, ACTC1, MYL2, MYL3, CSRP3	Less common collectively <10%	Contribute to a smaller proportion of cases	[15-17, 20]
Hypertrophic cardiomyopathy	Nonsarcomeric gene variants are rare and under study	MYH6, MYLK2, TCAP	Rare	Their role in disease pathogenesis is less understood	[23-24]
Dilated cardiomyopathy	Familial DCM has a well-established genetic foundation	TTN	TTN: 15%-25% of cases	TTN isoforms affect ventricular compliance	[25,27-30]
Dilated cardiomyopathy	Nonfamilial DCM may involve de novo or polygenic mutations	LMNA, SCN5A, FLNC	LMNA: ~6% of cases	LMNA mutations are linked to arrhythmias and conduction abnormalities	[25,28,35-38]
Arrhythmogenic right ventricular cardiomyopathy	Mutations in desmosomal protein genes lead to myocyte detachment and fibrosis, increasing arrhythmia risk	DSP, DSG2, DSC2, JUP, PKP2	PKP2: most common in ARVC	Complex inheritance patterns, with some cases showing compound or digenic heterozygosity	[39-47]

Advantages of utilizing CRISPR-Cas9 technique in somatic gene deletion in mice regarding cardiomyocytes

The CRISPR-Cas9 genome editing system has revolutionized cardiovascular research and genomic therapies. Its efficiency, high throughput, cost-effectiveness, and simplicity have made it the preferred tool for creating mutant mice to study gene function and uncover the genetic causes of cardiomyopathies [48]. However, deploying CRISPR components in vivo to induce loss-of-function mutations can be challenging. To address this, CRISPR-Cas9-AAV-based somatic mutagenesis (CASAAV) has been developed, utilizing Rosa26-Cas9-GFP mice with a Cre-inducible Cas9 allele. This method employs vectors that express sgRNAs targeting specific genes and Cre recombinase, both controlled by the cardiac troponin T (cTnT) promoter [49]. These approaches are effective in eliminating specific genes in cardiomyocytes.

Somatic gene editing offers a more efficient way to study gene function in adult cardiomyocytes compared to traditional conditional and inducible gene deletion methods. However, its efficiency depends on several factors, including the concentration of the AAV9 virus, sgRNA levels, and Cas9 expression. Variability in these factors can result in incomplete infection of cardiomyocytes by the AAV9 virus, leading to incomplete gene deletions and, ultimately, mosaic gene editing [50]. Despite this, mosaicism, which results in gene editing in a subset of cells, can provide valuable insights into cardiomyocyte maturation. Compared to stem cell-based homologous recombination, CRISPR-Cas9 offers more uniform gene editing across cells [51]. A major challenge in studying cardiomyocyte maturation is embryonic or fetal mortality caused by complete gene deletion in cardiac cells, which hinders functional analyses. To overcome this, CASAAV has been used to identify RYR2 as a key regulator of T-tubule development in cardiomyocytes through mosaic gene inactivation [52]. Additionally, a live CRISPR screen was conducted to explore cardiomyocyte development. This screen involved CASAAV in newborn R26Cas9-GFP/+ and Myh7YFP/+ pups infected with AAV vectors containing sgRNAs targeting over 2,000 genes [53]. This approach revealed several transcriptional and genetic regulators of maturation, showcasing the advantages of mosaic gene editing with CRISPR-Cas9 over traditional methods like shRNA knockdown in mice [54]. Mosaic gene suppression can also be achieved using the Cre-LoxP system. By administering small doses of AAV-cTnT-Cre to mice with floxed alleles, where loxP sites flank specific exons of a target gene, researchers can induce targeted gene knockout. For instance, depletion of the serum response factor (SRF) using this method has demonstrated SRF's crucial role in cardiomyocyte maturation. This role would have been obscured in conventional knockouts due to fatality (Table 2) [55].

**Table 2:** Summary of CRISPR-Cas9 techniques for somatic gene deletion in mice cardiomyocytes CRISPR: clustered regularly interspaced short palindromic repeats; Cas9: CRISPR-associated protein 9; CASAAV: CRISPR-Cas9-AAV-based somatic mutagenesis; SRF: serum response factor

Technique	Advantages	Limitations	Specific examples	Applications
CRISPR-Cas9	High efficiency in gene editing; high throughput; cost-effective; simplicity in design	Variability in gene editing efficiency, potential mosaicism; incomplete deletions	Used for creating mutant mice to study cardiomyopathies; evaluating gene functions in cardiomyocytes (e.g., RYR2)	Investigating gene function in adult cardiomyocytes; studying genetic causes of cardiomyopathies
CASAAV (CRISPR-Cas9-AAV)	Effective for in vivo somatic mutagenesis; high specificity with Cre-inducible Cas9; reduced fetal mortality	Dependence on AAV9 virus concentration; risk of incomplete infection; mosaicism	AAV9sgRNAcTnTCre vectors used for specific gene targeting; identified RYR2 as a key regulator in cardiomyocyte development	Targeted gene deletion in cardiomyocytes; overcoming challenges of gene function studies in embryonic stages
Cre-LoxP system	Precise control over gene deletion; reduced impact on fetal mortality compared to conventional methods	Complexity in vector design; requires floxed alleles; limited to specific gene targets	Revealed the role of SRF in cardiomyocyte maturation; effective for studying gene function in adult mice (e.g., SRF)	Gene function analysis in adult cardiomyocytes; exploring gene roles in mature cardiac cells
Live CRISPR screening	Allows screening of large gene sets; identifies transcriptional and genetic regulators of maturation	Requires sophisticated vector systems; potential for off-target effects	Conducted in R26Cas9GFP/+;Myh7YFP/+ pups; targeted over 2000 genes to find maturation regulators	High-throughput screening for gene regulators; identifying genetic pathways in cardiomyocyte development

Utilization of CRISPR-Cas9 technique for correcting genetic mutations involving HCM

HCM is a genetic disorder characterized by the abnormal thickening of the heart muscle, particularly the ventricles. This condition often leads to impaired cardiac function, arrhythmias, and an increased risk of SCD. HCM is primarily inherited in an autosomal dominant pattern, with mutations in genes encoding sarcomere proteins, such as MYH7, being the most common genetic cause [56]. In vivo, preclinical studies of gene editing approaches attributed to this disease have shown promising therapeutic advancements in genetic correction of its pathological manifestations. Recent studies have utilized distinct but complimentary CRISPR-Cas9 adenine base editing (ABE) system strategies. This approach targets a well-characterized missense mutation within the MYH7 gene, which encodes for beta myosin heavy chain [57]. The replacement of arginine by glutamine in this variation leads to increased contractility of cardiomyocytes and is characterized by the hallmarks of HCM. To address this issue, it is necessary to genetically correct or delete the mutated alleles [57]. According to an experimental in vivo study by Chai et al., only approximately 70% of transcriptional correction of p.Arg403Gln within ventricles is enough to prevent the pathological manifestations of HCM at the molecular level within the mice [58]. However, compared to the ventricles, the atria demonstrated lower editing efficiency following a single-dose injection, likely due to reduced coronary blood flow and perfusion [59]. This was addressed by an additional dose, which hindered atrial remodeling [58]. This study utilized a specific form of ABE and gRNA that enhanced the accuracy in editing the p. Arg403Gln variant and thus significantly reduced the editing bystander genes within the editing window. Furthermore, the researchers developed a unique humanized mouse model of the Myh7 p. Arg403Gln, and to assess the efficiency of the genetic editing in vivo, heterozygous and homozygous versions of the mice were used, which ultimately allowed for the use of gRNA-specific for human use. Within control, the genetically engineered heterozygous mice developed ventricular hypertrophy and showed myocardial fibrosis by nine months of age. The homozygous population, on the other hand, exhibited severe cardiomyopathy and fibrosis, which led to their mortality by one week postnatally. To remedy this, the main objective was to deliver the ABE and gRNA through AAV9 vectors through intrathoracic injections to cardiomyocytes controlled by the cTnT promoter [58]. Injection of high doses in homozygous mice increased their life span by up to two weeks due to an approximately 35% correction at the transcriptional level. In heterozygous mice, the correction value was like that of homozygous mice while effectively preventing ventricular hypertrophy and remodeling for up to 16 weeks. This was achieved with low bystander and off-target editing at five out of 16 potential loci, with neither causing adverse outcomes in treated mice [58].

**Figure 1:**
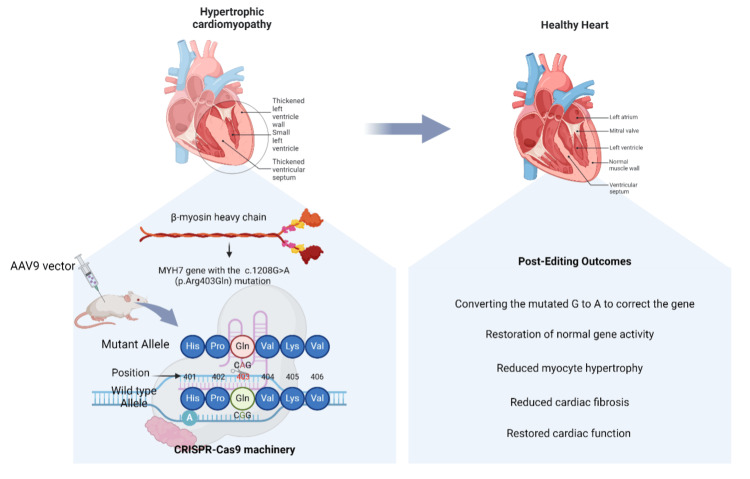
Utilization of CRISPR-Cas9 for genetic correction of hypertrophic cardiomyopathy (HCM) CRISPR: clustered regularly interspaced short palindromic repeats; Cas9: CRISPR-associated protein 9 The figure illustrates the process of correcting a specific genetic mutation associated with HCM, which is characterized by the abnormal thickening of the heart muscle, particularly in the left ventricle and septum. The MYH7 gene mutation c.1208G>A (p.Arg403Gln) leads to the substitution of arginine (Arg) with glutamine (Gln) in the β-myosin heavy chain protein, resulting in hypertrophy of cardiomyocytes. The CRISPR-Cas9 system, delivered via AAV9 vectors, targets and corrects this mutation by converting the mutated G back to A, thereby restoring normal gene function. Postediting outcomes include reduced myocyte hypertrophy, decreased cardiac fibrosis, and restoring normal cardiac structure and function, leading to a healthier heart. Image created by the authors

Moreover, in another study conducted by Reichart et al., a similar technique was used to rectify identical pathological mutations in presymptomatic mice [60]. This study utilized AAV9 vectors to deliver ABE and gRNA to cardiomyocytes; however, the expression of the siRNAs was under the control of cardiomyocyte-specific chicken troponin T (TnnT2) with a nonhumanized mouse model that had a pathological mutation in the orthologous position of the Myh6 gene in mice, which precluded the testing of human-specific gRNAs and resorted in the use of mouse-specific gRNAs [60]. Treatment in the male mice with a 129SvEv background that commonly develops cardiomyopathy around 20-25 weeks at 10-13 days postnatally resulted in 68% gene correction in ventricular cardiomyocytes and 26%-39% correction in atrial cardiomyocytes. Examinations conducted at 32-34 weeks demonstrated the reversal of cardiac hypertrophy and decreased formation of scar tissue in the heart. However, bystander editing was present within this treatment, as it was shown to increase with consecutive AAV injections. As the literature suggests, this phenomenon most likely occurred due to using an ABE with a wide editing range and a less specific PAM (Figure 1) [54]. Additionally, as an alternative approach, *Staphylococcus aureus *Cas9 was used instead of ABE to selectively introduce a double-stranded break and deactivate the p. Arg403Gln variation. Functional testing demonstrated the effective editing and correction of hypertrophic phenotypes. However, greater doses resulted in decreased contractile cardiac performance, indicating accidental editing of the normal alleles in cardiomyocytes [60]. This emphasizes the limited range of effectiveness of this approach [61].

Utilization of the CRISPR-Cas9 technique for correcting genetic mutations involving DCM

The utilization of CRISPR-Cas9 gene editing has become a prominent method for investigating DCM, offering a focused strategy for examining the genetic origins of this complex illness. Liu and Olson conducted several enlightening experiments applying the CRISPR-Cas9 system in several animal models to eliminate or repair specific mutations in crucial genes such as Titin (TTN) and MYH7, which are vital for the structural integrity and proper functioning of cardiomyocytes. The results were promising, showing significant improvements in heart function and muscle structure, suggesting the potential of CRISPR-Cas9 in reducing the effects of DCM. Moreover, this research employed advanced mice animal models, such as Myh6-Cas9 and Rosa26-Cas9-GFP mice, to delete particular genes in cardiomyocytes and to investigate genes associated with the maturation of cardiac cells. This study addressed both the underlying mechanisms of DCM and identified potential novel targets for its treatment. While the study yielded favorable results, it also reported certain challenges, including the efficacy of AAV9-based delivery techniques and the possibility of mosaicism in genetic editing [62]. All of these variables can potentially affect the treatment's overall efficacy. Nevertheless, the findings significantly support the potential of CRISPR-Cas9 in developing accurate therapies for DCM and other related cardiac conditions. The significance of CRISPR-Cas9 is further demonstrated by its use in repairing mutations in important genes, including TTN, LMNA, and SCN5A, all of which are essential for preserving cardiomyocyte structure and function [63]. One of the most frequent causes of DCM is mutations in TTN, especially those that result in truncating variants (TTNtv). These mutations impair the functionality of titin, a protein that is crucial for the flexibility and structural integrity of the cardiac muscle [64]. These frameshift mutations were corrected using CRISPR-Cas9 in the Myh6-Cas9 mouse model, subsequently restoring normal gene function. The implementation of this technique resulted in the creation of functioning titin proteins, which greatly improved the ability of the heart muscles to contract and decreased the expansion of the ventricles [62].

Additionally, the LMNA gene, responsible for producing Lamin A/C, is often associated with DCM. LMNA mutations can result in both abnormalities in the nuclear envelope's structure and disturbances in electrical conduction [63]. These can lead to the gradual weakening of the heart muscle and the development of arrhythmias. The CRISPR-Cas9 technique has been employed to rectify these genetic mutations in human induced pluripotent stem cells (iPSCs) obtained from individuals with LMNA mutations [65]. The studies demonstrate that by either knocking out the abnormal gene or making correct genetic modifications, the nuclear structure and function in the abnormal heart muscle cells returned back to normal [65]. This emphasizes the potential of gene editing to reverse the harmful effects of LMNA mutations and provides a promising approach for treating DCM [66].

**Figure 2:**
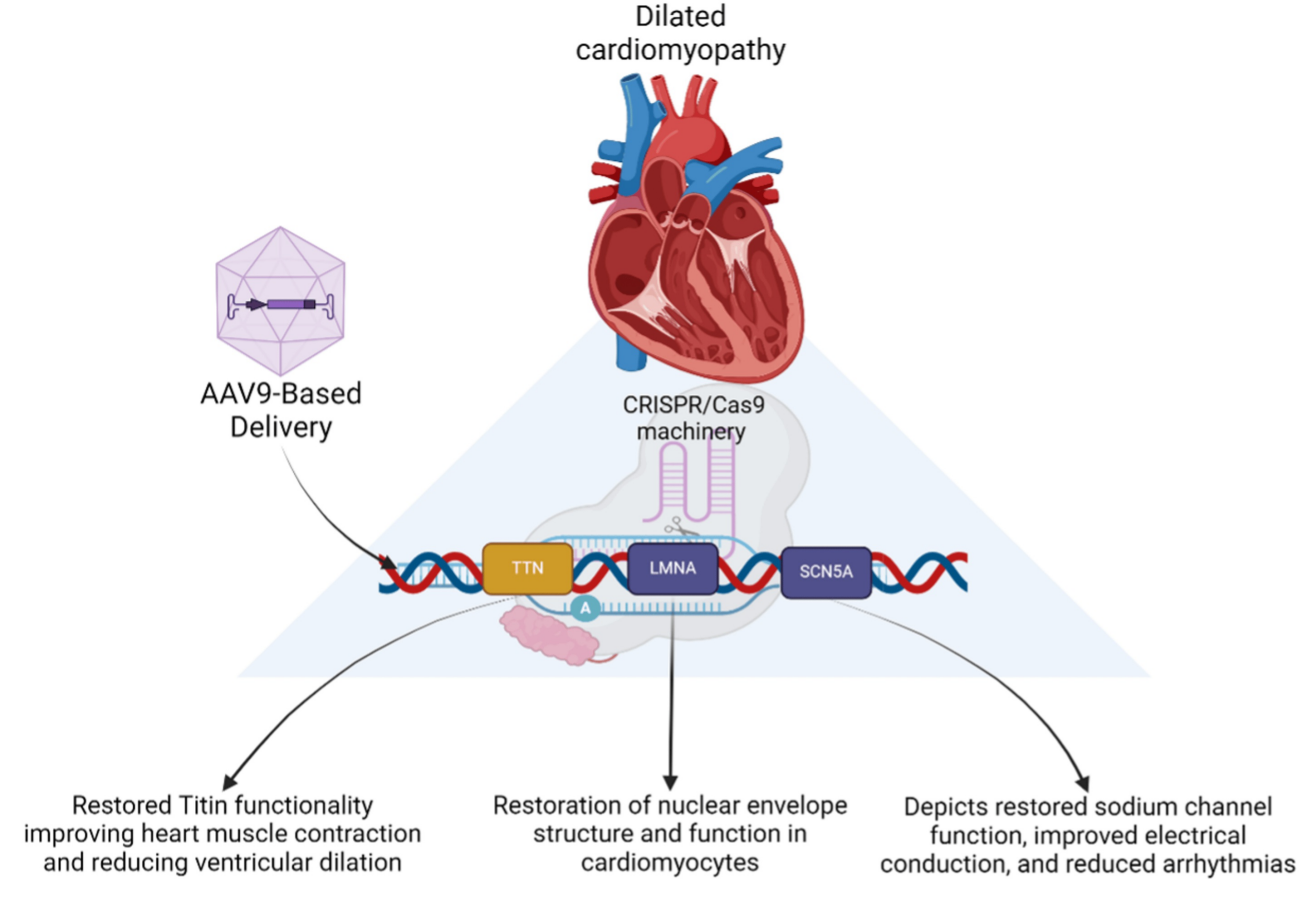
Application of CRISPR-Cas9 gene editing in the study and treatment of DCM DCM: dilated cardiomyopathy; CRISPR: clustered regularly interspaced short palindromic repeats; Cas9: CRISPR-associated protein 9 The figure illustrates the use of CRISPR-Cas9 technology to target and edit mutations in crucial genes associated with DCM, such as TTN, LMNA, and SCN5A. The process includes the delivery of the CRISPR-Cas9 machinery via AAV9-based vectors, which enables the correction of genetic defects, resulting in restored heart muscle function, improved electrical conduction, and reduced ventricular dilation. This approach demonstrates the potential of gene editing in addressing the genetic causes of DCM and improving cardiac health. Image created by the authors

The SCN5A gene encodes the cardiac sodium channel Na_v1.5, a crucial element in the conduction of electrical signals in the heart. People with SCN5A mutations may have issues with the passage of electrical signals and irregular heartbeats, which could worsen DCM and intensify its symptoms [67]. CRISPR-Cas9 has successfully repaired SCN5A mutations in both patient-derived cardiomyocytes and animal models. The edited genes in the cells exhibited a decreased risk of arrhythmias, improved electrical flow, and repaired sodium channel activity, demonstrating CRISPR-Cas9's ability to target structural and electrophysiological issues in relation to DCM (Figure 2) [68].

Utilization of the CRISPR-Cas9 technique for correcting genetic mutations involving AVRC

ARVC is a hereditary condition that predominantly impacts the right ventricle of the heart, resulting in irregular heart rhythms and a heightened likelihood of SCD [41]. The condition is strongly linked to abnormalities in genes that encode desmosomal proteins, which are crucial for preserving the structural integrity of cardiomyocytes [69]. Five specific desmosomal genes cause ARVC: DSP, DSG2, DSC2, JUP, and PKP2. Among these genes, PKP2 is the most frequently altered. The latest developments in CRISPR-Cas9 genome editing offer a hopeful method for rectifying these mutations and potentially addressing ARVC at its genetic source. Researchers conducted a study to investigate the use of CRISPR-Cas9 for correcting mutations in PKP2. They used a mouse model that expressed the human PKP2 c.2013delC mutation, which is referred to as Pkp2 c.1755delA in mice. The model demonstrated the typical attributes of ARVC, such as decreased expression of desmosomal proteins, compromised heart function, and the presence of fibrosis. The researchers successfully rectified the mutation in induced pluripotent stem cell-derived cardiomyocytes (iPSC-CMs) using CRISPR-Cas9-mediated gene editing. The repaired cells exhibited normalized PKP2 expression, enhanced cell-cell adhesion, and improved electrical conductivity, resulting in the restoration of cardiac function [70].

CRISPR-Cas9 has proven to be useful in targeting additional desmosomal genes, including DSP, DSG2, and DSC2. Specifically, iPSCs carrying mutations in DSG2 were modified utilizing CRISPR-Cas9 technology to either disable the mutated gene or make accurate modifications. After undergoing development into cardiomyocytes, these modified cells exhibited repaired desmosomal integrity and enhanced resistance to stress-induced separation, a prevalent problem in ARVC. Moreover, the normalization of the expression of desmosomal proteins resulted in improved cell adhesion and electrical stability, both of which are crucial for preventing arrhythmias [71].

**Figure 3:**
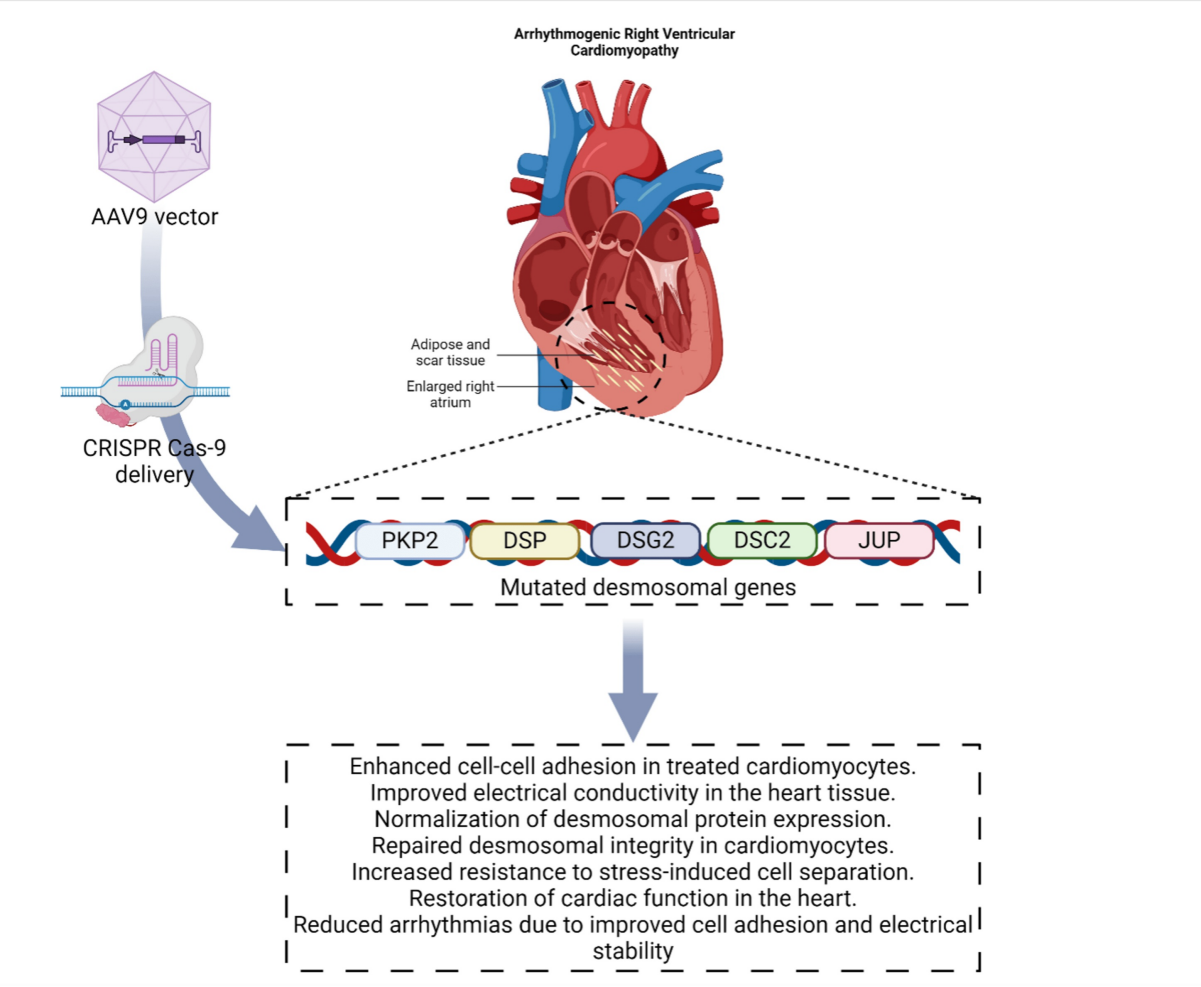
Schematic representation of ARVC highlighting the genetic basis and potential therapeutic intervention using CRISPR-Cas9 AVRC: arrhythmogenic right ventricular cardiomyopathy; PKP2: plakophilin 2; DSP: desmoplakin; DSG2: desmoglein-2; DSC2: desmoscollin-2; JUP: junctional-plakophilin; CRISPR: clustered regularly interspaced short palindromic repeats; Cas9: CRISPR-associated protein 9 ARVC predominantly affects the right ventricle, leading to irregular heart rhythms and an increased risk of sudden cardiac death. The condition is associated with mutations in desmosomal genes (PKP2, DSP, DSG2, DSC2, and JUP), which compromise the structural integrity of cardiomyocytes. The figure illustrates the delivery of CRISPR-Cas9 via an AAV9 vector to target and correct these mutations. Outcomes of successful gene editing include enhanced cell-cell adhesion, improved electrical conductivity, normalization of desmosomal protein expression, repaired desmosomal integrity, increased resistance to stress-induced separation, restoration of cardiac function, and reduced arrhythmias. Image created by the authors

An obstacle in using CRISPR-Cas9 for ARVC therapy is guaranteeing the effective and accurate transportation of the editing apparatus to the desired cells. Research has emphasized the significance of enhancing AAV9 vectors for transporting CRISPR components to the heart while also tackling challenges such as mosaicism in gene editing, which can impact the effectiveness of the therapeutic intervention. Although faced with these obstacles, the effective utilization of CRISPR-Cas9 in rectifying desmosomal gene alterations in both animal models and human iPSC-derived cardiomyocytes serves as compelling evidence for its promise as a therapeutic approach for ARVC (Figure 3) [70].

Discussion and future perspectives

CRISPR-CAS 9 technology is a significant advancement in the treatment of hereditary cardiomyopathies, marking a pivotal moment for cardiovascular medicine and a genuine breakthrough in this area. The gene editing technique, CRISPR-CAS 9, known for its accuracy and adaptability, has the potential to revolutionize the treatment of HCM, DCM, and ARVC. Despite the amazing pace of growth in recent years, there are still numerous hurdles that need to be addressed before CRISPR-Cas9 can be widely used for therapeutic purposes in these disorders [48].

The exciting aspect of CRISPR-Cas9 for HCM is its ability to specifically and efficiently target pathogenic mutations in sarcomeric genes like MYH7. Even a partial gene correction can lead to significant decreases in disease pathology. For instance, correcting approximately 70% of the gene was enough to eliminate the molecular signs of HCM in animal models. Nevertheless, the differences in editing efficiency reported among various areas of the heart and between the ventricles and atria necessitate additional refinement of the delivery strategies [56-58]. The relatively poor editing efficiency seen in the atria, likely attributed to blood flow and perfusion variations, presents a crucial area that warrants additional exploration. While the probability of off-target effects is extremely low in certain trials, it nevertheless poses a significant risk that should be thoroughly examined through long-term studies in different genetic backgrounds.

CRISPR-Cas9 holds great potential as a tool for correcting gene mutations that result in gene truncations, such as TTN [60,62]. TTN plays a critical role in maintaining the structural integrity of cardiomyocytes in DCM. Furthermore, the expansion of the dilative processes after surgery appeared to be a result of the gene therapy and subsequent recovery of functioning titin proteins in the mice models [62]. However, the occurrence of mosaicism, which refers to the creation of inconsistent modifications, is becoming a growing concern for the consistency and replicability of this treatment. The efficacy of CRISPR component distribution by AAV vectors is a crucial factor that will ultimately decide the effectiveness of therapy [54]. To address these issues, creating more efficient and precise delivery technologies that can uniformly repair genes in the myocardium is crucial. A significant issue with the illness is its correlation with mutations in desmosomal proteins responsible for cell adhesion and transmission of signals in the heart. CRISPR-Cas9 has demonstrated potential in rectifying mutations in various target genes, leading to the restoration of normal cardiac function in cardiomyocytes produced from iPSCs, such as PKP2. Regrettably, achieving efficient delivery of CRISPR components to the heart while simultaneously minimizing off-target effects and mosaicism is challenging [70,71].

Furthermore, additional research is necessary to examine the durability of these adjustments over an extended period and their potential to halt disease advancement in living organisms. The implemented characteristic is pertinent to the interaction between the modified and unmodified cells within the heart tissue and the resulting vulnerability to arrhythmias. To summarize, while CRISPR-Cas9 offers a unique chance to edit and rectify genetic abnormalities that cause cardiomyopathies, the practical application of using CRISPR for therapeutic correction of these conditions is still far from being used in clinical practice. Currently, the limited capacity to enhance productivity in editing, unintended consequences, and the difficulty in delivering the desired results pose significant obstacles that need to be addressed. Continued research is necessary to optimize the delivery methods and improve the specificity of CRISPR-based medicines. The future of gene therapy in treating cardiomyopathies has several potential advancements, but its realization depends on the dedication of the scientific community.

## Conclusions

This study explored the potential of CRISPR-Cas9 as a therapeutic approach for targeting genetic cardiomyopathies at their root cause. Preclinical studies have demonstrated significant progress in using CRISPR-Cas9 to treat various cardiomyopathies. However, there are some limitations, including variability in editing efficiency between various areas of the heart, delivery challenges, off-target effects, and mosaicism. Future studies should focus on optimizing delivery systems, minimizing off-target effects, addressing the variability in editing efficiency, and developing strategies to reduce the occurrence of mosaicism.
